# Low avidity circulating SARS-CoV-2 reactive CD8+ T cells with proinflammatory TEMRA phenotype are associated with post-acute sequelae of COVID-19

**DOI:** 10.3389/fmicb.2023.1196721

**Published:** 2023-06-02

**Authors:** Krystallenia Paniskaki, Margarethe J. Konik, Moritz Anft, Harald Heidecke, Toni L. Meister, Stephanie Pfaender, Adalbert Krawczyk, Markus Zettler, Jasmin Jäger, Anja Gaeckler, Sebastian Dolff, Timm H. Westhoff, Hana Rohn, Ulrik Stervbo, Carmen Scheibenbogen, Oliver Witzke, Nina Babel

**Affiliations:** ^1^Department of Infectious Diseases, West German Centre of Infectious Diseases, University Hospital Essen, University Duisburg-Essen, Essen, Germany; ^2^Center for Translational Medicine and Immune Diagnostics Laboratory, Medical Department I, Marien Hospital Herne, University Hospital of the Ruhr-University Bochum, Bochum, Germany; ^3^CellTrend GmbH, Luckenwalde, Germany; ^4^Department of Molecular and Medical Virology, Ruhr-University Bochum, Bochum, Germany; ^5^Department of Nephrology, University Hospital Essen, University Duisburg-Essen, Essen, Germany; ^6^Medical Department I, Marien Hospital Herne, University Hospital of the Ruhr-University Bochum, Herne, Germany; ^7^Institute for Medical Immunology, Charité-Universitätsmedizin Berlin, Campus Virchow, Berlin, Germany; ^8^Berlin Institute of Health at Charité – University Clinic Berlin, BIH Center for Regenerative Therapies (BCRT) Berlin, Berlin, Germany

**Keywords:** post-acute sequelae of COVID-19, PASC, long COVID, T cells, BMI

## Abstract

The role of adaptive SARS-CoV-2 specific immunity in post-acute sequelae of COVID-19 (PASC) is not well explored, although a growing population of convalescent COVID-19 patients with manifestation of PASC is observed. We analyzed the SARS-CoV-2-specific immune response, via pseudovirus neutralizing assay and multiparametric flow cytometry in 40 post-acute sequelae of COVID-19 patients with non-specific PASC manifestation and 15 COVID-19 convalescent healthy donors. Although frequencies of SARS-CoV-2-reactive CD4+ T cells were similar between the studied cohorts, a stronger SARS-CoV-2 reactive CD8+ T cell response, characterized by IFN*γ* production and predominant T_EMRA_ phenotype but low functional TCR avidity was detected in PASC patients compared to controls. Of interest, high avidity SARS-CoV-2-reactive CD4+ and CD8+ T cells were comparable between the groups demonstrating sufficient cellular antiviral response in PASC. In line with the cellular immunity, neutralizing capacity in PASC patients was not inferior compared to controls. In conclusion, our data suggest that PASC may be driven by an inflammatory response triggered by an expanded population of low avidity SARS-CoV-2 reactive pro-inflammatory CD8+ T cells. These pro-inflammatory T cells with TEMRA phenotype are known to be activated by a low or even without TCR stimulation and lead to a tissue damage. Further studies including animal models are required for a better understanding of underlying immunopathogensis. Summary: A CD8+ driven persistent inflammatory response triggered by SARS-CoV-2 may be responsible for the observed sequelae in PASC patients.

## Introduction

Post-infectious Myalgic Encephalomyelitis/Chronic Fatigue Syndrome (ME/CFS) is already described as a sequelae after numerous primarily viral infections (Epstein–Barr virus, cytomegalovirus, etc.) and SARS-CoV-2 virus is lately adding to the list (O’Neal and Hanson, 2021). Post-acute sequelae of COVID-19 presents as a chronic multisystemic disease characterized by a variety of respiratory, cardiovascular, gastroenterological and neurological symptoms (Carruthers et al., 2011; Evans et al., 2021; Nalbandian et al., 2021; Seeßle et al., 2021). Growing body of literature suggests that a combination of virus and host factors, residual inflammation (Komaroff and Bateman, 2021; O’Neal and Hanson, 2021; Peluso and Deeks, 2022), microvascular dysregulation/endothelial injury (Fogarty et al., 2021; Mehta et al., 2021; Wang et al., 2022), autoimmune phenomena and abnormal cellular energy metabolism (Balinas et al., 2019; Proal and Van Elzakker, 2021) contribute to PASC. Emerging epidemiologic studies (Mazza et al., 2020, 2021) draw attention to concentration disorders, posttraumatic stress disorder, sleep disorders as psychiatric/neurological sequelae after COVID-19 disease. To this end, several studies demonstrated anatomical changes in structures of the brain of COVID-19 survivors with post-acute COVID-19 (Benedetti et al., 2021; Tu et al., 2021; Hanson et al., 2022).

Similar to ME/CFS, immune alterations have been also demonstrated to be associated with PASC. Thus, distinct immune changes have newly been described in PASC with primarily lung sequelae. Cheon et al. (2021) showed that functional SARS-CoV-2-specific memory T and B cells are abundant in bronchial lavage fluid of COVID-19 adults with primarily lung sequelae compared to those of blood. Vijayakumar et al. (2022) demonstrate distinct immune and proteomic changes in post-COVID-19 airways, where increased bronchoalveolar lavage cytotoxic T cells are linked to epithelial damage and airway disease. Few studies approach experimentally the immune mechanisms responsible for system specific PASC separately- gastroenterological, respiratory, cardiovascular or neurological PASC. However, data on SARS-CoV-2 specific cellular and humoral immunity in PASC as a disease entity, which affects a significant proportion of the general population are currently missing. To address these knowledge gaps and the contribution of immunity, including humoral and cellular response in PASC pathogenesis, we performed an immune profiling of 40 patients with PASC. As control we used 15 healthy COVID-19 convalescent adults.

## Materials and methods

### Study participants

We used peripheral blood mononuclear cells (PBMCs) and serum samples from 40 convalescent COVID-19 patients with post COVID-19 syndrome (further referred as PASC) and 15 convalescent COVID-19 patients without clinical manifestation of post COVID-19 syndrome (further referred as control). The clinical criteria of post-acute COVID-19 syndrome as defined by Nalbandian et al. (2021) and NICE guidelines^1^ were applied to set the diagnosis of post COVID-19 syndrome and therefore recruitment of the study participants. Cognitive/psychiatric impairment/symptomatic was based on ICD 10 psychiatric relevant diagnoses. Demographic and clinical characteristics are provided in Tables 1, 2.

**Table 1:** Clinical Symptoms and comorbidities of the study cohorts.

		PASC *N* (%)	Control *N* (%)	*p* value
PASC type	Ongoing COVID-19	0 (0)	N/A	N/A
	Post-acute COVID-19 Syndrome	40 (100)	N/A	N/A
Post-acute COVID-19 symptoms	Shortness of breath/exercise intolerance	28 (70)	N/A	N/A
	Cognitive disturbances (brain fog)	23 (57.5)	N/A	N/A
	Fatigue	17 (42.5)	N/A	N/A
	Decline of life quality	16 (40)	N/A	N/A
	Dyspnea	11 (27.5)	N/A	N/A
	Muscular weakness	9 (22.5)	N/A	N/A
	Anxiety/depression	7 (17.5)	N/A	N/A
	Sleep disturbances	6 (15)	N/A	N/A
	Chest pain	4 (10)	N/A	N/A
	Joint pain	3 (7.5)	N/A	N/A
	Cough	3 (7.5)	N/A	N/A
	Headaches	3 (7.5)	N/A	N/A
	Smell/taste disturbance	3 (7.5)	N/A	N/A
	Posttraumatic stress disorder	2 (5)	N/A	N/A
	Thromboembolism	2 (5)	N/A	N/A
	Persistent oxygen requirement	0 (0)	N/A	N/A
	Palpitations	0 (0)	N/A	N/A
	Hair loss	0 (0)	N/A	N/A
Comorbidities prior to COVID-19	Obesity	33 (82.5)	3 (20)	0
	Arterial hypertension	9 (22.5)	0 (0)	0.0494
	Asthma bronchiale	4 (10)	0 (0)	0.5665
	Malignity	3 (7.5)	0 (0)	0.5519
	Autoimmune disease	3 (7.5)	0 (0)	0.5519
	Depression	3 (7.5)	0 (0)	0.5519
	Diabetes	3 (7.5)	0 (0)	0.5519
	Coronary heart disease	1 (2.5)	0 (0)	1

**Table 2:** Demographic and clinical characteristics of the study cohorts.

	PASC (*N* = 40)	Control (*N* = 15)	*p* value
Age years -median (range)	51.5 (19–68)	30 (19–50)	0.0002****
Female gender *N* (%)	25 (63)	10 (70)	1
BMI -median (range)	29 (17.6–52.5)	22 (17.9–35)	0.0003
Time since COVID-19 Diagnosis (months)	10 (2–16)	8 (2–12)	0.0773
Duration of PASC symptoms (months)*	10 (2–16)	N/A	N/A
COVID-19 severity *N* (%)			
*Moderate*	38 (96)	15 (100)	1
*Severe*	1 (2)	0 (0–0)	1
*Critical*	1 (2)	0 (0–0)	1
Time last vaccination up to recruitment (months)**	4 (1–8)	4 (1–8)	0.8132
Variant of concern N*** (%)			
*WT*	25 (63)	8 (53)	0.5533
*Alpha*	13 (32)	1 (7)	0.0808
*Delta*	1 (2.5.)	4 (27)	0.0166
*Omicron*	1 (2.5)	2 (13)	0.1774

* Months from SARS-CoV-2 Diagnosis (positive PCR) till the time point of study recruitment.

** Months from last vaccination up to study recruitment.

*** The viral variants that the study participants were infected with were determined relying on temporal viral spreading defined by epidemiologcal trends in Germany at the recruitment timepoint.

**** Bivariate regression analysis excluded age interference on T cell frequencies (Supplementary Table S2).

### Preparation of PBMCs

As previously described, peripheral blood was collected in S-Monovette K3 EDTA blood collection tubes (Sarstedt) (Anft et al., 2020; Paniskaki et al., 2022). Collected blood was prediluted in PBS/BSA (Gibco) at a 1:1 ratio and underlaid with 15 mL of Ficoll-Paque Plus (GE Healthcare). Tubes were centrifuged at 800 g for 20 min at room temperature. Isolated PBMCs were washed twice with PBS/BSA and stored at −80°C until use. The cryopreserved PBMCs were thawed by incubating cryovials 2–3 min at 37°C in bead bath, washed twice in 37°C RPMI 1640 media (Life Technologies) supplemented with 1% penicillin–streptomycin-glutamine (Sigma-Aldrich), and 10% fetal calf serum (PAN-Biotech) medium, and incubated overnight at 37°C.

### Flow cytometry – measurement of SARS-CoV-2 reactive T cells

As previously described, PBMCs were plated in 96-U-Well plates in RPMI 1640 media (Life Technologies) (Anft et al., 2020; Paniskaki et al., 2022). Each well was stimulated with the wildtype (WT) Spike (S) SARS-CoV-2 protein (Miltenyi Biotec) or left untreated as a control for 16 h. The proteins were dissolved per manufacturer’s directions. As a positive control, cells were stimulated with staphylococcal enterotoxin B (1 μg/mL, Sigma-Aldrich). After 2 h, brefeldin A (1 μg/mL, Sigma-Aldrich) was added. Detailed listing of the antibody panels for general phenotyping and T cell activation *ex vivo* is in Supplementary Table S2. The PBMCs stimulated overnight were stained with optimal concentrations of antibodies for 10 min at room temperature in the dark. Stained cells were washed twice with PBS/BSA before preparation for intracellular staining using the Intracellular Fixation & Permeabilization Buffer Set (Thermo Fisher Scientific) as per the manufacturer’s instructions. Fixed and permeabilized cells were stained for 30 min at room temperature in the dark with an optimal dilution of antibodies against the intracellular antigen. All samples were immediately acquired on a CytoFLEX flow cytometer (Beckman Coulter) (gating strategy, Supplementary Figure S2). Quality control was performed daily using the recommended CytoFLEX daily QC fluorospheres (Beckman Coulter). No modification to the compensation matrices was required throughout the study. Antigen-reactive responses were considered positive after the non-reactive background was subtracted, and more than 0.01% were detectable. Negative values were set to zero.

### SARS-CoV-2 neutralization assay

As previously described (Paniskaki et al., 2022), for the virus neutralization assay, sera were incubated for 30 min at 56°C in order to inactivate complement factors. Single cycle VSV∗ΔG(FLuc) pseudoviruses bearing the SARS-CoV-2 WT-S (D614G) protein in the envelope were incubated with quadruplicates of two-fold serial dilutions of immune sera in 96-well plates prior to infection of Vero E6 cells (1×10^4^ cells / well) in DMEM +10% FBS (Life Technologies). At 18 h post infection, firefly luciferase (FLuc) reporter activity was determined using a CentroXS LB960 (Berthold) and the reciprocal antibody dilution causing 50% inhibition of the luciferase reporter was calculated (PVND50).

### Statistics

Flow cytometry data were analyzed using FlowJo version 10.6.2 (BD Biosciences); gating strategies are presented in Supplementary Figure S1. For the analysis of anti-SARS-CoV-2 reactive T cells, a threshold of 0.01% was employed to define a detectable response. Single stains and fluorescence-minus-one controls were used for gating. Gates of each study participant were adjusted according to the negative control. CD4+ T cells expressing CD154 and CD137 and CD8+ T cells expressing CD137 were defined as reactive T cells. Statistical analysis was performed using GraphPad Prism v7. Categorical variables are summarized as numbers and frequencies; quantitative variables are reported as median and interquartile range. Normality Tests were performed with Shapiro–Wilk test. All applied statistical tests are two-sided. Frequencies of SARS-CoV-2-protein reactive T cells in the PASC study group and the control group were compared using exact two-tailed Mann–Whitney test. The age between the two cohorts was compared using unpaired two-tailed *t*-test, and gender was compared using two-tailed Fisher’s exact test. Correlational relationships were explored with Spearman’s test. *p* values below 0.05 were considered significant; only significant *p* values are reported in the figures. *p* values were not corrected for multiple testing, as this study was of an exploratory nature.

## Results

### Characterization of the study groups

Our study group comprised 40 convalescent COVID-19 patients with PASC (further referred as PASC) and 15 convalescent COVID-19 patients without clinical manifestation of post COVID-19 syndrome (further referred as control). The clinical criteria of PASC as defined by Nalbandian et al. (2021) and NICE guidelines were applied to set the diagnosis of post COVID-19 syndrome and therefore recruitment of the study participants. All study participants had a negative SARS-CoV-2 nasal swab tested via PCR on recruitment. During the acute phase of COVID-19 disease, 100% (*n* = 15) and 96% (*n* = 38) of the control and PASC group respectively, presented moderate COVID-19 disease severity without need for hospitalization, whereas only 4% (*n* = 2) were severely or critically ill and hospitalized. The demographic characteristics and clinical symptoms of PASC are summarized in Table 1.

The median COVID-19 convalescence time for the control and PASC study groups at the timepoint of the recruitment was 8 (range 2–12) and 10 (range 2–16) months, respectively. All control individuals (*n* = 15) and 82.5% (*n* = 33) of the PASC study group received at least two COVID-19 mRNA vaccinations (either prior or after the infection). The latest COVID-19 vaccination took place in a median time of 4 (range 1–8) months before the study recruitment for both study groups.12.5% (*n* = 7) of the PASC study group were not vaccinated against SARS-CoV-2 neither before nor after the infection. As we studied convalescent subjects, a direct molecular sequencing of the viral variant responsible for SARS-CoV-2 infection was not possible. The viral variants that the study participants were infected with were determined relying on temporal viral spreading defined by epidemiologcal trends in Germany at the recruitment timepoint. The majority of the PASC patients and control study participants were infected with the WT (Table 2).

The median age of the PASC study group was 51.5 years (range 19–68 years), whereas the control cohort was significantly younger, with a median age of 30 years (range 19–50 years, *p* = 0.0002 two tailed unpaired *t* test).

The PASC and control cohorts comprised of 63% (*n* = 25) and 70% (*n* = 10) female participants, respectively and showed no statistical gender difference (Fisher’s exact test, *p* > 0.05). PASC patients (median BMI 29, range 17.6–52.5) showed significantly higher BMI compared to controls (median BMI 22, range 17.9–35) (Mann Whitney test, *p* = 0.0003). All PASC study subjects suffered from at least two symptoms. Regarding comorbidities, the PASC study group presented significantly higher blood hypertension rates (Fisher’s exact test, *p* = 0.0494) compared to the controls. The demographic and clinical characteristics of the study cohorts are presented in Tables 1, 2.

### PACS is associated with higher frequencies of WT S-reactive CD8+ T cells

To explore the role of cellular immunity related to COVID-19, we analyzed SARS-CoV-2 reactive T cells. As the majority of the study participants were infected with the WT, we addressed the WT S-reactive CD4+ and CD8+ T cell response. WT S-reactive CD4+ and CD8+ T cells are further referred as S-reactive CD4+ and CD8+ T cells.

**Figure 1:**
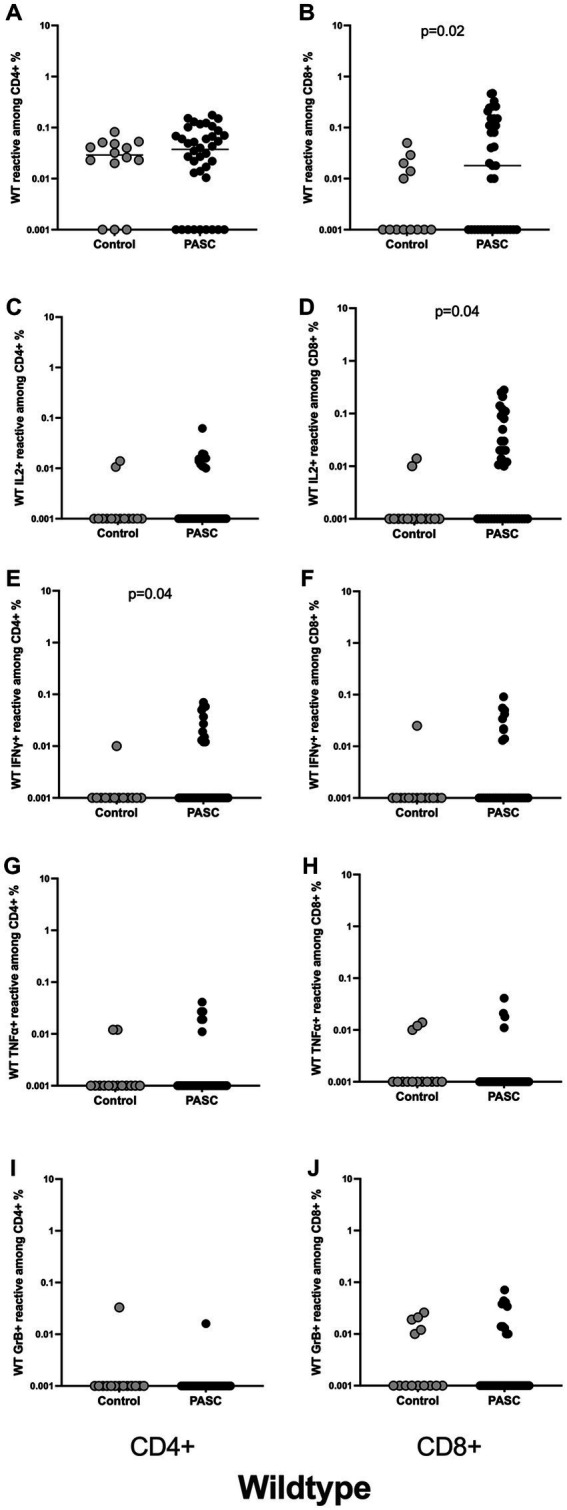
Higher frequencies of SARS-CoV-2 WT reactive CD8+ T cells among the PASC study group. Characterization of SARS-CoV-2 S-reactive T cells in PASC and control subjects. Blood samples of 40 PASC patients and 15 convalescent controls were stimulated with SARS-CoV-2 S-WT and analyzed by flow cytometry. **(A,B)** Frequencies of WT- reactive CD4+ and CD8+ T cells among PASC and controls. **(C,D)** IL2 producing SARS-CoV-2 reactive CD4+ and CD8+ T cells. **(E,F)** IFN*γ* producing SARS-CoV-2 reactive CD4+ and CD8+ T cells. **(G,H)** TNF*α* producing SARS-CoV-2 reactive CD4+ and CD8+ T cells. **(I,J)** GrB producing SARS-CoV-2 reactive CD4+ and CD8+ T cells. SARS-CoV-2 S-reactive CD4+ and CD8+ T cells are defined as CD4 + CD154 + CD137+ and CD8 + CD137+ cells, respectively. Antigen-reactive responses were considered positive after the unstimulated background was subtracted, and more than 0.01% were detectable. Scatterplots show line at median. Unpaired data were compared with Mann–Whitney-test. *p* < 0.05 was considered significant, only significant *p* values are documented in the figures.

The magnitude of CD4+ T cells directed against spike protein did not differ significantly between the analyzed groups. Thus, the frequencies of S-reactive CD4+ T cells were comparable between the control and PASC study groups (Figure 1A). Analyzing functionality of SARS-CoV-2-reactive CD4+ T cells as defined by T cell cytokine production, we observed a significantly higher frequencies of IFN*γ*-producing S-reactive CD4+ T cells in PACS patients as compared to control group (Figure 1E). The frequencies of S-reactive T cells producing other cytokines including IL2, TNF*α* and GrB showed similar frequencies between the two cohorts (Figures 1C,G,I).

In contrast to the CD4+ T cell data, we observed statistically significant differences for S-reactive CD8+ T cells. Thus, the frequencies of S-reactive CD8+ T cells were significantly higher among the PASC compared to the controls (Figure 1B). Interestingly, S-reactive CD8+ IL2 producing T cells (Figure 1D) showed significantly higher frequencies among the PASC patients. S-reactive CD8+ T cells producing IFN*γ*, TNF*α*, and GrB showed similar frequencies between the two cohorts (Figures 1F,H,J).

**Figure 2:**
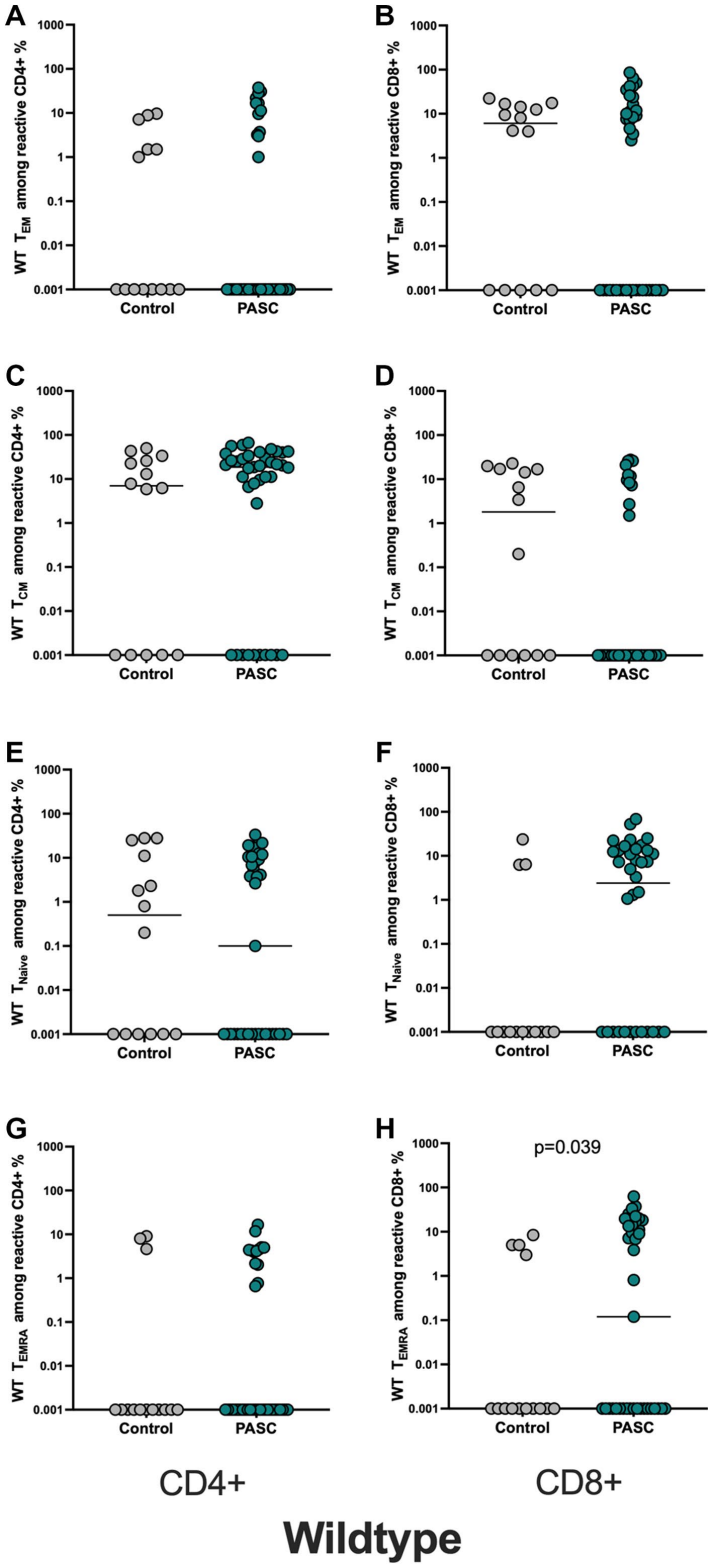
Higher frequencies of WT-reactive CD8+ T_EMRA_ cells among the PASC study group. Evaluation of the memory subsets was performed using the markers CCR7 and CD45RA (T_CM_ = CD45RA-CCR7+, T_NAIVE_ = CD45RA + CCR7+, T_EM_ = CD45RA-CCR7- T_EMRA_ = CD45RA + CCR7-). Frequencies of the memory subsets of WT-reactive CD4+ and CD8+ T cells in PASC subjects were compared to controls. **(A,B)** WT reactive CD4+ and CD8+ T_EM_ cells. **(C,D)** WT reactive CD4+ and CD8+ T_CM_ cells. **(E,F)** WT reactive CD4+ and CD8+ T_NAIVE_ cells. **(G,H)** WT reactive CD4+ and CD8+ T_EM_ cells.

The analysis of phenotypic differentiation of SARS-CoV-2 reactive T cells, defined by the expression or absence of CD45RA and CCR7 (gating strategy, Supplementary Figure S2) showed significantly higher frequencies of S-reactive CD8+ T_EMRA_ cells in PASC patients versus control, whereas other subsets showed no differences between the two cohorts (Figures 2A–H).

### SARS-CoV-2 specific T cell frequencies are independent of BMI and age

As aging is related to immunosenescence and chronic inflammation (DiCarlo et al., 2009; Fulop et al., 2018) and our PASC study group was significantly older compared to controls we performed a bivariate regression correlation analysis to exclude potential bias of age on the immune response. The bivariate regression analysis showed no correlational relationship between T cell frequencies and the age of the participants (Supplementary Table S2).

Furthermore, to exclude potential bias of all other demographic or clinical variable on the frequencies of T cells including BMI or different time interval between the last antigenic contact and blood collection, we performed a correlation analysis for all explored variables. The found significant differences in T cell immunity appeared to be independent from other variables (Supplementary Table S2). In particular, we found no correlation between the duration since the last antigenic contact or BMI and S-reactive CD4+ or CD8+ T cells (Supplementary Table S2).

### PASC is associated with a significantly higher number of S-reactive CD8+ T cell with low TCR avidity

**Figure 3:**
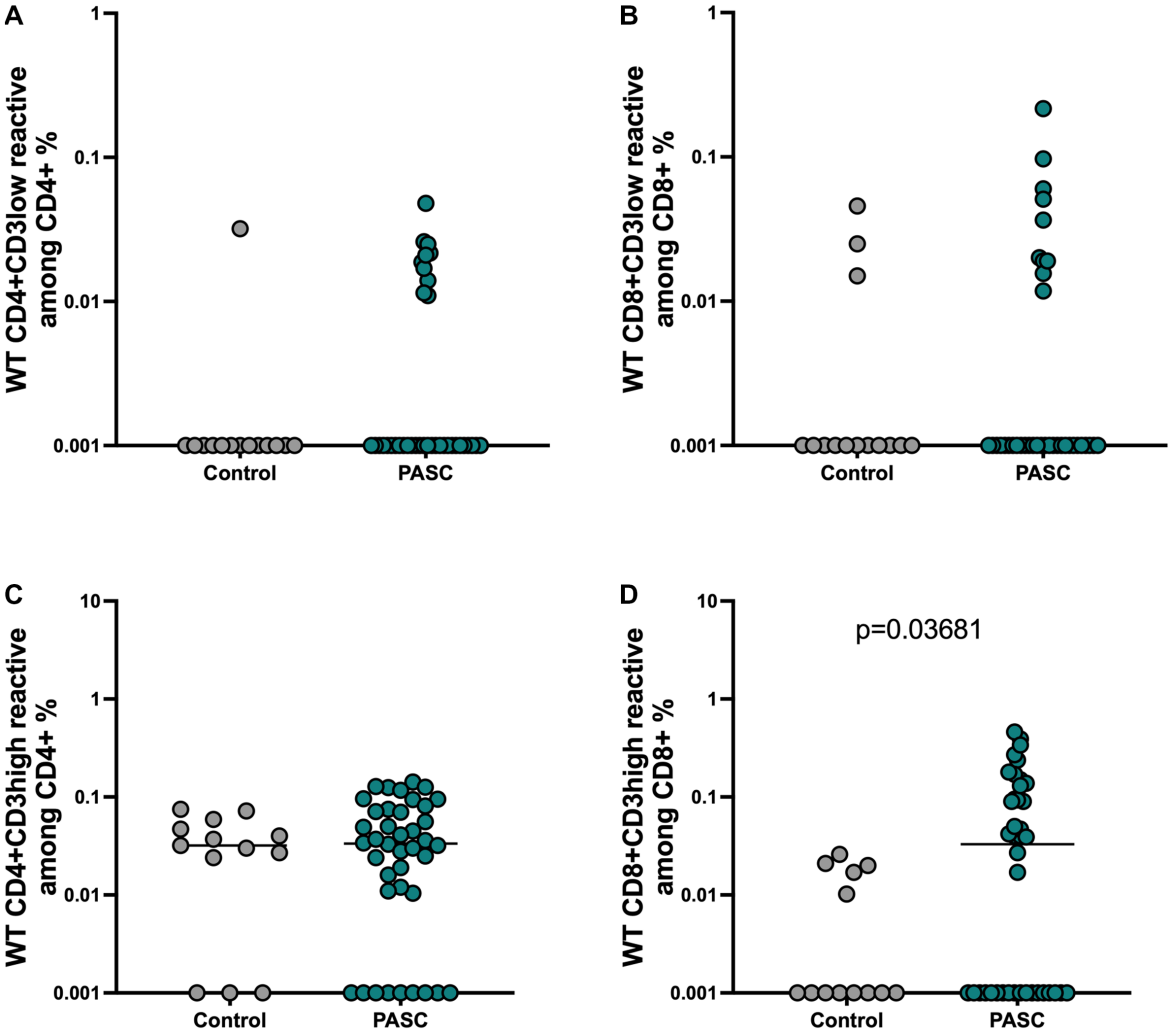
Presence of low avidity CD8 T cell response among the PASC patients. The maximum functional avidity of SARS-CoV-2 reactive T cells was approached by determining the CD3low + cells among CD4 + CD154 + CD137+ and CD8 + CD137+ cells, while low TCR avidity was determined by detecting CD3high + cells among CD4 + CD154 + CD137+ and CD8 + CD137+ cells. **(A,B)** Frequencies of WT reactive CD4 + CD3low + and CD8 + CD3low + T cells. **(C,D)** Frequencies of WT reactive CD4 + CD3high + and CD8 + CD3high + T cells.

Bacher et al. suggested the important role of functional avidity for viral clearance especially in context of SARS-CoV-2 infection (Bacher et al., 2020). Therefore, we also performed an analysis of the avidity of SARS-CoV-2- reactive T cells. Strong TCR activation, which is characteristic of T cells with high TCR avidity, blocks recycling of the TCR-CD3 complex and can be detected by reduced CD3 surface expression, a phenomenon known as high functional avidity (Loyal et al., 2021; Paniskaki et al., 2022). Therefore, analyzing the frequencies of CD3low T cells within activated CD4+ or CD8+ T cells will demonstrate the T cells with high avidity, whereas CD3high T cells within activated CD4+ or CD8+ S-reactive T cells correspond to T cells with a low TCR avidity. Applying this method as performed before (Loyal et al., 2021; Paniskaki et al., 2022) (gating strategy, Supplementary Figure S1), we detected similar frequencies of reactive CD4+ and CD8 + CD3low reactive T cells among PASC and controls (Figures 3A,B), indicating that both study groups can achieve similar maximum functional avidity status among the S-reactive T cell populations. However, the analysis of the CD3high subsets addressing T cells with low functional avidity, demonstrated S-reactive CD8 + CD3high T cells with significantly higher frequencies among the PASC study group compared to control patients (Figures 3C,D).

### Similar neutralizing antibody response in PASC patients and controls

High neutralizing antibodies (NAb) titers are considered to protect effectively against SARS-CoV-2 infection, and their waning is related with high risk of reinfection or vaccine breakthrough infection (Kent et al., 2022). Currently it is unclear, whether the NAbs play a role in PASC pathogenesis, therefore we measured the titers of WT Nabs in PASC and control individuals. We found similar titers of spike IgGs and WT NAbs among the two study groups indicating that neutralizing capacity is not impaired among the PSC group (Supplementary Figure S3).

## Discussion

According to Taquet et al., PASC symptoms show no clinical excess when compared to other respiratory infections (Taquet et al., 2022). However, its occurrence is more frequent, as COVID-19 incidence is higher compared to other respiratory infections (Xue et al., 2022). Beside the burden of PASC on the health care system, PASC has been correlated to reduced working hours and inability to work in 22% of PASC patients (Davis et al., 2021). Despite its benignity in the majority of the cases, PASC is a public health and social issue requiring interdisciplinary attention. Here, we performed an immune profiling including S-reactive cellular immune responses and neutralizing capacity in 40 patients with PASC. We demonstrated significantly higher frequencies of S-reactive CD8+ T cells in PASC group. Even more interestingly, comparing TCR avidity of spike reactive T cells, we found a significantly higher number of CD8+ T cells with functional low TCR avidity, whereas the number of CD4+ and CD8 T cells with high TCR avidity did not differ between the groups.

While SARS-CoV-2 T cells with the high functional TCR avidity have been associated with better specificity to the cognate antigen (SARS-CoV-2) and a better functional activity, SARS-CoV-2 reactive T cells with a low TCR avidity have been demonstrated to be cross-reactive (Loyal et al., 2021). Following these observations, we tempt to hypothesize that the patients with PASC have a sufficient antiviral cellular response. Comparable titers of neutralizing antibodies in PASC and control groups underscore our assumption. On the other hand, the increased number of SARS-CoV-2 reactive CD8+ T cells with a low TCR avidity in PASC group might indicate their involvement in pathogenesis of PASC. Bacher et al. have already demonstrated the relevance of low avidity S-reactive T cells in immunopathogenesis of acute SARS-CoV-2 infection (Bacher et al., 2020).

The expansion of cytotoxic CD8+ T cells independently of their specificity is already demonstrated to be an important pathogenic component of gastrointestinal and pulmonary PASC by other groups (Cheon et al., 2021; Phetsouphanh et al., 2022; Su et al., 2022). It has been proven that upregulation of CD8+ T cells contributes to autoimmunity driven demyelination and axonal damage (Bitsch et al., 2000; Friese and Fugger, 2009; Gilmore et al., 2020; Lind et al., 2021), while highly differentiated effector CD8+ T_EMRA_ cells have been associated with neuroinflammation and degeneration (Northfield et al., 2007; Benveniste and Allenbach, 2019). In the frame of SARS-CoV-2 infection independent groups demonstrated that the majority of long-lived antigen specific CD8 memory T cells present TEMRA phenotype (Dan et al., 2021; Jung et al., 2021; Adamo et al., 2022). In line with these findings, the phenotypic characterization of our S-reactive T cells revealed significantly higher number of CD8 T cells with TEMRA phenotype. TEMRA cells are terminally differentiated and carry the highest levels of cytotoxic and exhaustion genes and molecules compared to T cells with other developmental phenotypes (Xiong et al., 2023). Furthermore CD8+ TEMRA cells are known to proliferate poorly but have strong effector activity such as killing and cytokine release (Reinke et al., 2013). In line with it, we observed an increased number of IFN*γ* producing T cells in PASC group. Furthermore, TEMRA cells are known to be activated even without the need for TCR cross-linking by cognate antigen suggesting therefore that the low TCR avidity found in T cells of PASC patients can be compensated by the ability of TEMRA cells to be activated via cytokine receptors and provide tissue damage as reported previously (Reinke et al., 2013).

An important issue was to exclude that the found alterations in cellular immunity were not biased by any clinical or demographic variables. Thus, obesity has been correlated with increased risk for PASC development (Reinke et al., 2013; Scherer et al., 2022) and is linked to adipose chronic tissue inflammation characterized by infiltration and activation of immune cells that overproduce cytokines and chemokines (Tilg and Moschen, 2006; Sell et al., 2012; Han and Levings, 2013; Liu and Nikolajczyk, 2019; Larabee et al., 2020; Aminian et al., 2021). We demonstrate that the CD8+ driven SARS-CoV-2 specific T cell response is not correlated to BMI. Our results indicate that although obesity predisposes PASC manifestation, it was not associated with the observed persistent cellular inflammation. In agreement to our findings, Littlefield et al. found no correlation of antigen specific T cell frequencies with age or number of comorbidities-including metabolic syndrome (Littlefield et al., 2022).

There are some limitations of this study that should be addressed. We could not explore correlations between T cell response and the clinical severity of PASC symptoms, therefore future studies addressing the impact of T cell immunity in PASC symptomatology would be of interest. Littlefield et al., demonstrated that elevated frequencies of SARS-CoV-2-specific T cells in individuals with pulmonary PASC are associated with increased systemic inflammation and decreased lung function (Littlefield et al., 2022), while Finlay et al. (2022) showed that persistent post–COVID-19 smell loss is associated with immune cell infiltration in olfactory epithelium.

In summary, our data suggest that an expanded population of pro-inflammatory low avidity SARS-CoV-2 reactive and terminally differentiated CD8+ T cells contribute, among other factors, in immunopathogenesis of PASC. This antigen specific persistent inflammation detected in circulation may reflect/represent an inflammatory response in diseased tissues, as a result of antigenic persistence (Brodin et al., 2022; Swank et al., 2023) or in the frame of autoimmunity. Further studies including animal models are required for a better understanding of the underlying mechanisms.

## Data availability statement

The raw data supporting the conclusions of this article will be made available by the authors, without undue reservation.

## Ethics statement

The studies involving human participants were reviewed and approved by Ethics Committee of University Hospital Essen (20-9753-BO). The patients/participants provided their written informed consent to participate in this study.

## Author contributions

KP, NB, MA, and US participated in research design. KP, MK, MZ, HR, SD, and AG participated in data curation and sample acquisition. KP, CS, and NB participated in the writing of the paper. NB, OW, TW, US, and HH participated in funding acquisition and project administration. KP, MA, TM, SP, HH, JJ, and AK participated in the performance of the research. NB, TW, OW, and US contributed new reagents or analytic tools. KP, NB, and MA participated in data analysis. All authors contributed to the article and approved the submitted version.

## Funding

This work was supported by grants of Mercator Foundation, EFRE grant for COVID.DataNet. NRW, AiF grant for EpiCov, and BMBF for NoChro (FKZ 13GW0338B).

## Conflict of interest

Author HH was employed by company CellTrend GmbH.

The remaining authors declare that the research was conducted in the absence of any commercial or financial relationships that could be construed as a potential conflict of interest.

## Publisher’s note

All claims expressed in this article are solely those of the authors and do not necessarily represent those of their affiliated organizations, or those of the publisher, the editors and the reviewers. Any product that may be evaluated in this article, or claim that may be made by its manufacturer, is not guaranteed or endorsed by the publisher.

## Footnotes

^1^ https://www.nice.org.uk/guidance/ng188/resources/covid19-rapid-guideline-managing-the-longterm-effects-of-covid19-pdf-51035515742
