# Supplementary material for: Low avidity circulating SARS-CoV-2 reactive CD8+ T cells with proinflammatory TEMRA phenotype are associated with post-acute sequelae of COVID-19
Source: Front Microbiol. 2023 Jun 2;14:1196721. doi: 10.3389/fmicb.2023.1196721 (PMC10272838; doi:10.3389/fmicb.2023.1196721)
Supplement: Supplementary file 1 [file Data_Sheet_1.PDF]

Low avidity circulating SARS-CoV-2 reactive CD8+ T cells with proinflammatory TEMRA phenotype are associated with post-acute sequelae of COVID-19

Supplemental Table of Contents

Supplementary Tables

Table S1: Fluorochrome coupled antibodies and fluorescent dye for analysis of SARS-CoV-2 reactive T cells
Table S2: Bivariate correlations - T cell frequencies

Supplementary Figures

Figure S1. Flow cytometry gating strategy for identification and quantification of SARS-CoV-2 reactive T cells.
Figure S2. Flow cytometry gating strategy for identification and quantification of memory SARS-CoV-2 reactive T cells.
Figure S3: PASC humoral immunity is not inferior compared to controls.

Table S1: Fluorochrome coupled antibodies and fluorescent dye for analysis of SARS-CoV-2 reactive T cells

Antibodies or fluorescent dye	Fluorochrome	Source	Cat. Nr.
Fixable Viability-Dye	eFluor780	eBioscience	65-0865-14
anti CCR7 (clone G043H7)	PerCP-Cy5.5	BioLegend	353220
anti CD4 (clone OKT4)	A700	BioLegend	317426
anti CD8 (clone RPA-T8)	V500	BD Biosciences	560775
anti CD45RA (clone HI100)	BV605	BioLegend	304134
anti Granzyme B (clone GB11)	FITC	BioLegend	515403
anti IL2 (clone MQ1-17H12)	PE	BioLegend	500307
anti CD185(CXCR5) (clone MP4-25D2)	PE-Dazzle594	BioLegend	356927
anti CD137 (4-1BB) (clone 4B4-1)	PE-Cy7	BioLegend	309818
anti CD154 (CD40L) (clone 24-31)	A647	BioLegend	310818
anti TNFα (clone MAb11)	eFluor450	eBioscience	48-7349-42
anti IFNγ (clone 4S.B3)	BV650	BioLegend	502538
anti CD3 (clone OKT3)	BV785	BioLegend	317330

Table S2: Bivariate correlations - T cell frequencies

	Correlation with age		Correlation with time since last antigenic exposure		Correlation with BMI	
	**Spearman r**	**p value**	**Spearman r**	**p value**	**Spearman r**	**p value**
WT CD137+ among CD8+	0,126493592386992	0,376420819441063	-0,034970941453606	0,807520849248596	0,0108580953664043	0,939719733162756
WTCD8high among CD8+	0,241049207827381	0,082076956652709	-0,0074177045361675	0,958377119738032	0,0773393195612975	0,585779704156355
WT CD4high among CD4+	0,244372998224411	0,0778187321987719	-0,274304342194713	0,0490819744674925	0,0848578047546361	0,549757439476105
WT CD137+ among CD8+	0,138022496022477	0,329173373245977	-0,034970941453606	0,807520849248596	0,0108580953664043	0,939719733162756
WT Temra among reactive CD8+	0,235416824587929	0,0929472803264865	0,0955198402778933	0,504922914580182	0,144583104265733	0,311412096918477
WT IFNg+ among reactive CD8+	0,0428660710757835	0,762853206835942	0,2639875617519	0,0612249144373214	0,114634499294818	0,423129144400351
WT IL2+ among reactive CD8+	-0,0207399689553892	0,883970286421597	-0,0233509719321996	0,870796194110983	-0,180674006434728	0,204528611454522

Supplementary figures

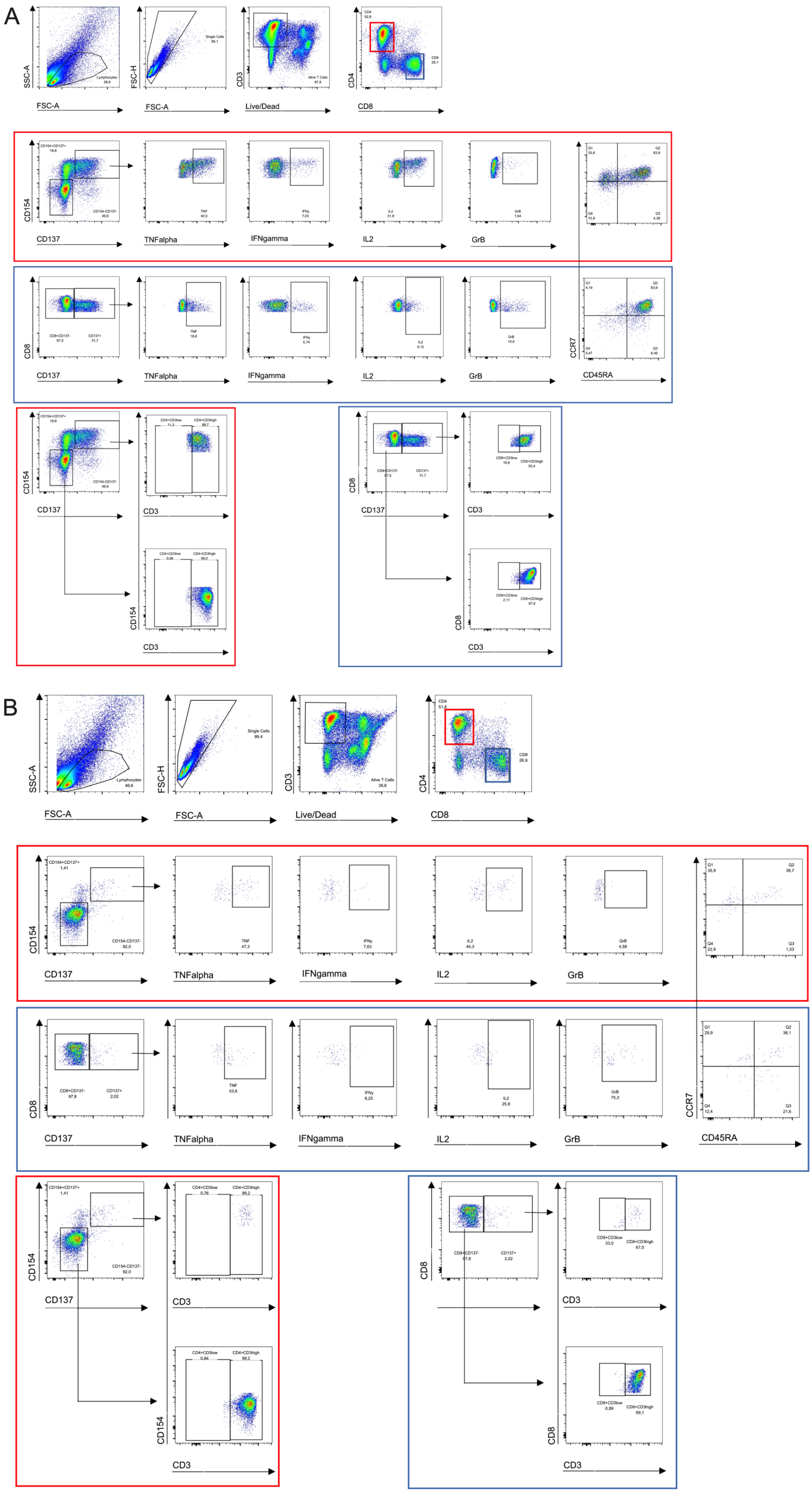

Figure S1. Flow cytometry gating strategy for identification and quantification of SARS-CoV-2 reactive T cells. PBMCs were stimulated for 16 h with SARS-CoV-2 peptides spanning *in silico* predicted immunodominant parts of the WT Spike SARS-CoV-2 (figure B) or with SEB (figure A) or left untreated. After 2 h, Brefeldin A was added to the culture to block secretion of cytokines and effector molecules. Living single lymphocytes were analyzed for expression of CD3, CD4, and CD8. CD4+ T cells (orange boxes) were analyzed for the expression of CD154 and CD137. CD8+ T cells (blue boxes) were analyzed for expression of CD137. Both CD4+ and CD8+ T cells were further analyzed for the production of cytokines IFNγ, TNFα, IL2 and GrB. Furthermore, CD4+CD154+CD137+ and CD8+CD137+ T cells were analyzed for the expression of CD3low and CD3high. Representative example of 40 patients with post COVID-19 syndrome and 15 healthy convalescent individuals. Plots of a PASC study subject are depicted.

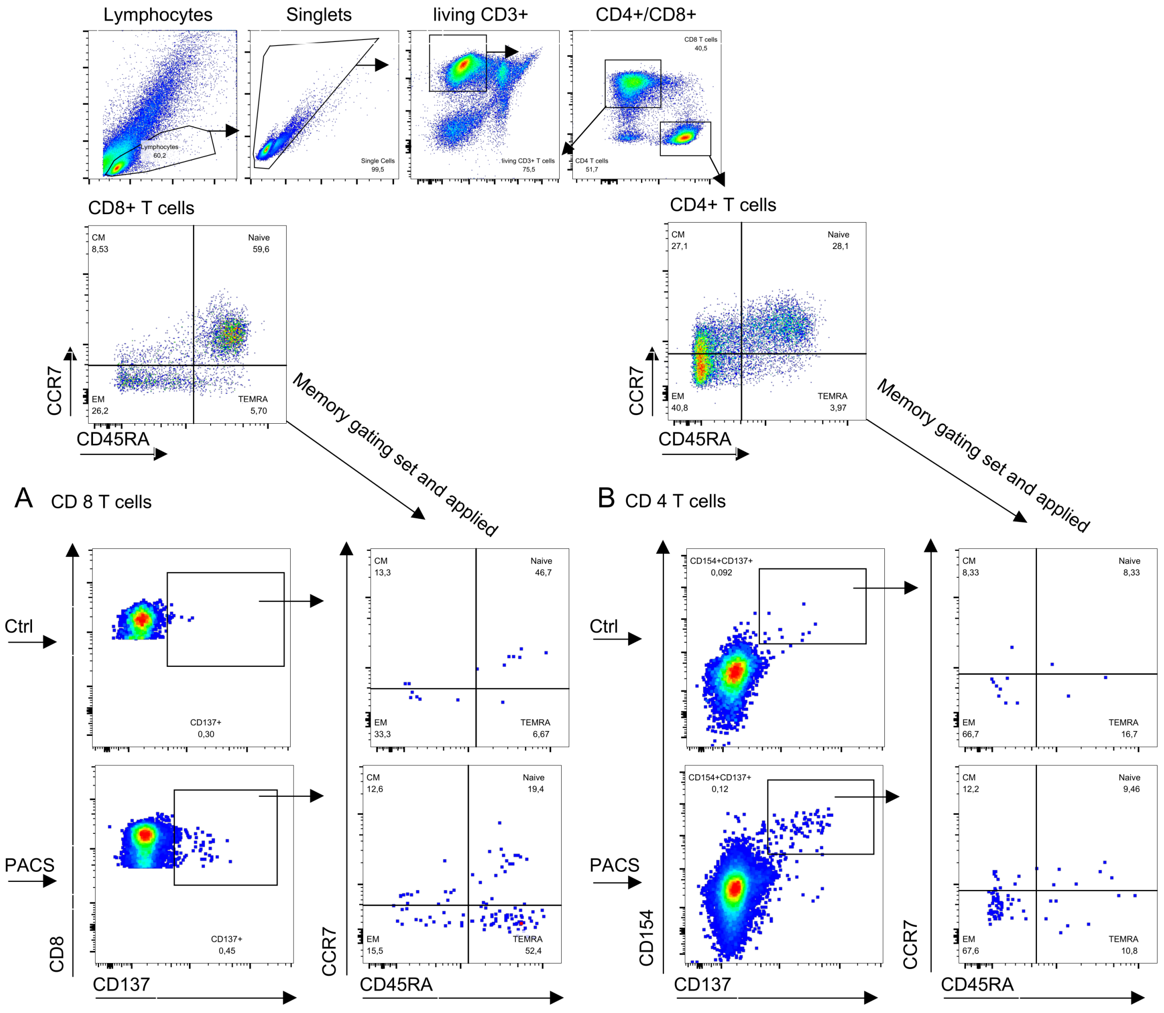

Figure S2. Flow cytometry gating strategy for identification and quantification of memory SARS-CoV-2 reactive T cells. CD4+CD154+CD137+ (B) and CD8+CD137+ (A) T cells were analyzed for the expression of CCR7 and CD45RA to evaluate the memory subsets (T_{CM}=CD45RA-CCR7+, T_{NAIVE}=CD45RA+CCR7+, T_{EM}=CD45RA-CCR7- T_{EMRA}=CD45RA+CCR7-). Pre-gating of the total memory CD4+ and CD8+ subsets was set and applied on SARS-COV-2 reactive CD4+ and CD8+ T cells. Plots of a PASC and a control study subject are depicted.

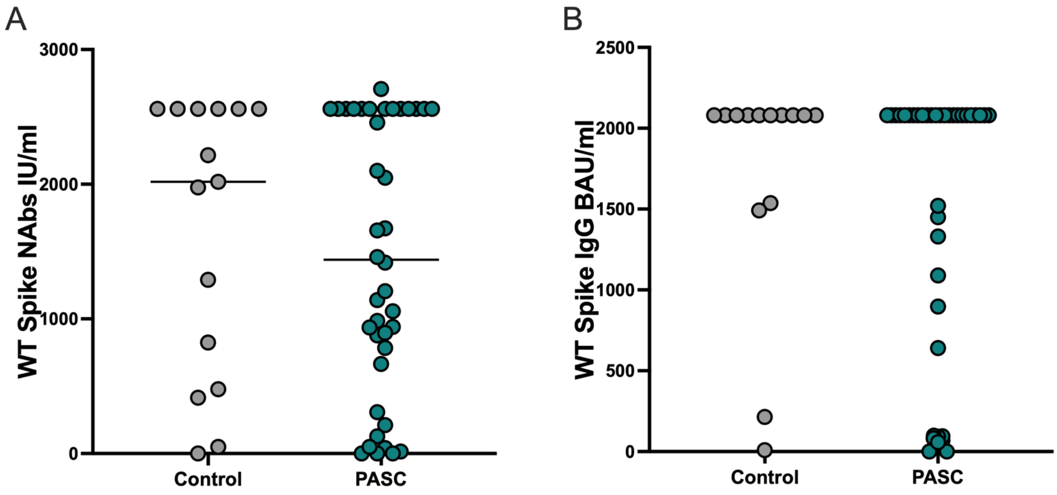

Figure S3: PASC humoral immunity is not inferior compared to controls. Analysis of WT Spike IgG and WT NAbs titers of both study groups. **(**A) Spike IgG titers. (B) WT NAbs titers. Scatterplots show line at median. Unpaired data were compared with Mann-Whitney-test. $P<0.05$ was considered significant, only significant p values are documented in the figures.
